# Application of 256-slice computed tomography with low radiation doses in neonates with hypoxic-ischemic encephalopathy

**DOI:** 10.3892/etm.2013.1322

**Published:** 2013-09-30

**Authors:** LIU QING, XIANGXING MA

**Affiliations:** Radiology Department, Qilu Hospital, Shandong University, Jinan, Shandong 250012, P.R. China

**Keywords:** low dose cranial computed tomography, 256-slice spiral computed tomography, brain computed tomography scan hypoxic-ischemic encephalopathy

## Abstract

Hypoxic-ischemic encephalopathy (HIE), an injury or disease with lack of oxygen in the brain, may occur at any stage in childhood but the exact mechanisms that cause HIE remain unknown. In this study, 150 newborns suspected of having neonatal HIE and scheduled for a brain CT scan were randomly assigned to three equally sized groups as follows: standard dose group (120 kV, 250 mAsec), low dose group 1 (120 kV, 150 mAsec) and low dose group 2 (120 kV, 50 mAsec). All other acquisition parameters were the same in all groups. The CT dose index (CTDI), dose length product (DLP) and the image noise were compared among the three groups. The image quality was evaluated by blinded readers. The DLP of low dose group 2 was 19.3% of that of the standard dose group without a significant difference (P>0.05). The image noise of the low dose group 1 was greater than that of the standard dose group with a significant difference (P<0.01). Low dose scanning is feasible in the screening of HIE in neonates and is beneficial in protecting newborns against unnecessary radiation damage.

## Introduction

Neonatal hypoxic-ischemic encephalopathy (HIE) may result from diffuse hypoxic-ischemic brain injury (1). It is one of the most common causes of cerebral palsy and other permanent neurological deficits in children (2,3). Therefore, a brain computed tomography (CT) scan is commonly used in the screening and diagnosis of HIE. However, newborns are far more radiosensitive than adults and suffer from potentially more serious injury. Therefore, the use of reduced radiation doses in neonatal CT scans is an important area of research in contemporary imaging technology (4). A 256-slice CT scanner has the fastest rotation speed and a novel detector, which guarantees lower radiation doses during examination (5). The aim of the present study was to assess the overall image quality and clinical value of 256-slice spiral CT with low radiation doses in the imaging of neonatal brains with suspected HIE.

## Patients and methods

### Clinical data

From March 2011 to March 2012, 150 newborns from Qilu Hospital (Jinan, China) were selected. There were 88 male and 62 female subjects, including 95 full-term and 55 premature cases. The gestational age was between 28–40 weeks and the birth weight ranged from 1,650 to 3,150 g. The newborns included 111 babies born via natural labor, while the remaining 39 cases were delivered by caesarean section. All parents/guardians of patients signed consent forms and all procedures were reviewed and approved by the Ethic Committee at the Qilu Hospital of Shandong University (Jinan, China).

### Methods

The CT scanner used in this study was a Philips Brilliance 256-slice spiral CT (Philips Medical Systems, Amsterdam, The Netherlands). This scanner automatically displays the CT dose-weighted index (CTDI) and dose-length product (DLP) during scanning.

### Acquisition parameters

**Figure 1:**
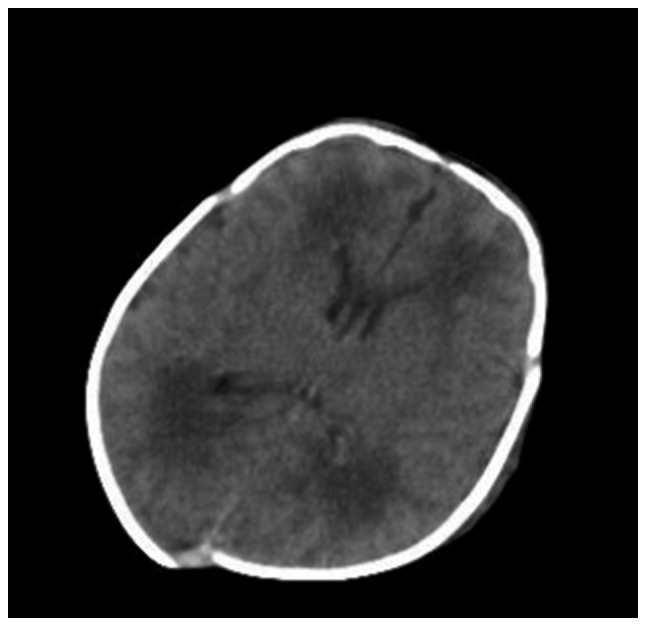
Standard dose computed tomography (120 kV, 250 mAsec).

**Figure 2:**
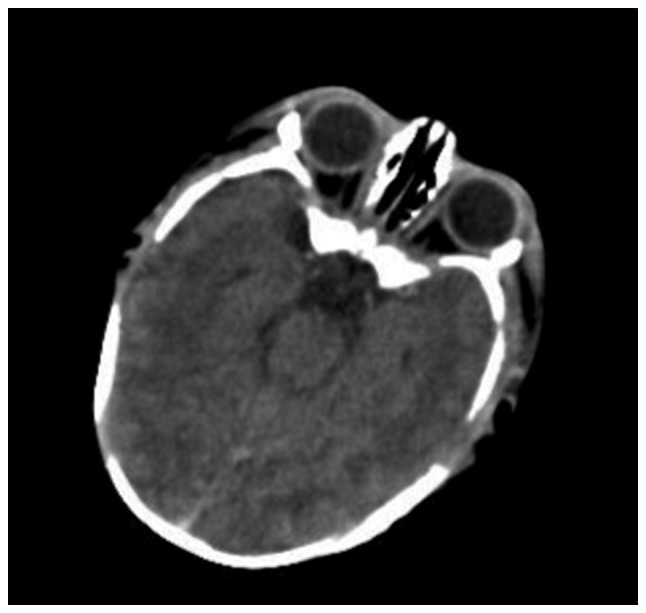
Low dose computed tomography (group 1; 120 kV, 150 mAsec).

**Figure 3:**
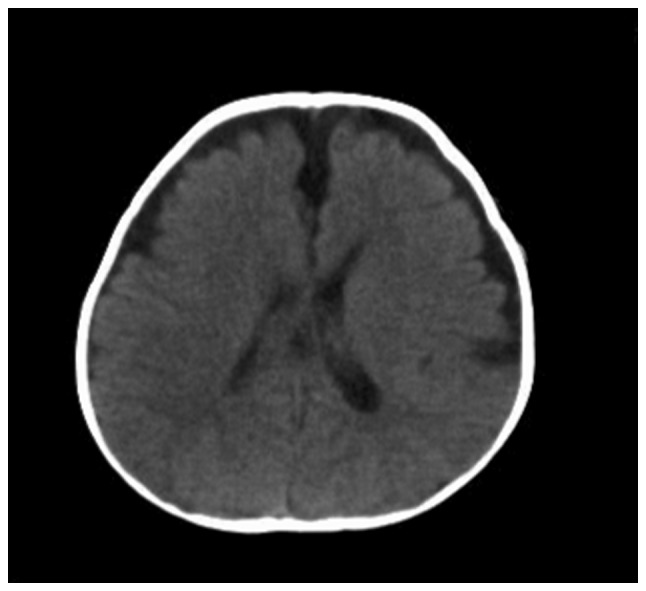
Low dose computed tomography (group 2; 120 kV, 50 mAsec).

All patients were randomly divided into three groups according to the radiation dose, as follows: standard dose group (Fig. 1) with 120 Kv, 250 mAsec; low dose group 1 (Fig. 2) with 120 Kv, 150 mAsec and low dose group 2 (Fig. 3) with 120 Kv, 50 mAsec. The slice thickness was 5 mm and the interlayer spacing was 5 mm. The newborn was placed in a supine position and a full brain scan was performed.

The CTDI and DLP were recorded from the scanner display. Since the DLP is related to the scanning range, the CTDI and DLP of the standard dose and low dose CT at the same scan length were also recorded. The CT values in the left basal ganglia were also measured by drawing the region of interest (ROI).

### Image quality assessment

The images were blindly assessed by two experienced physicians. The evaluation criteria were as follows: score 3, no image artifacts, sharp edges of the skull, good contrast between the gray and white matter, clear ventricle edge and clear lesions; score 2, some image artifacts and a lower signal-to-noise ratio, but the reduced image quality did not affect the overall diagnosis; and score 1, images had a greater amount of noise and the lesions were not clearly delineated, potentially making an accurate diagnosis challenging and complicated.

### Statistical analysis

The CTDI, DLP, signal noise and image quality were compared among the three groups. Data are presented as mean ± standard deviation (SD). SPSS 13.0 software (SPSS Inc., Chicago, IL, USA) was used to perform statistical analyses. P<0.05 was considered to indicate a statistically significant difference.

## Results

### Image quality rating

The image quality scores in the standard dose group, low dose group 1 and low dose group 2 were 2.55±0.29, 2.25±0.41 and 2.05±0.74, respectively. There was no statistical difference in the image quality rating among the three groups (P>0.05).

### Noise

The signal noise levels of the ROI in the standard dose group, low dose group 1 and low dose group 2 were 1.78±0.42, 1.95±0.35 and 2.36±0.49 HU, respectively. The measured noise levels of the groups were significantly different (P<0.05; Table I).

### Radiation dose

In the standard dose group, low dose group 1, and low dose group 2, the DLPs were 311.6, 109.7 and 60.2 mGy respectively. The DLP in low dose group 1 was 35.2% of the DLP in the standard dose group, and the DLP in low dose group 2 was 19.3% of the DLP in the standard dose group. A reduction in the radiation dose resulted in a decline in the DLP value. However, this did not affect the image quality. Although the image noise was relatively high in low dose group 2 (Fig. 3), intracranial structures and lesions were clearly delineated. The window and level settings may be adjusted to generate a diagnostic image.

## Discussion

Low dose CT scanning is currently used in a variety of clinical applications. The successful application of low dose CT scanning requires a good natural contrast between the skull, brain tissue and the ventricular system. In the present study, we reduced the radiation dose in multi-slice spiral CT scanning of the newborn brain without compromising the image quality. The appropriate dose ensures that the image has adequate contrast between normal brain structures and lesions in order to make a correct clinical diagnosis.

Multidetector CT (MDCT) achieves a larger volume of data acquisition, which broadens the applications of CT and improves the diagnostic level. 256-Slice CT scanning adopts conventional technology for the processing of high-speed data, but reduces the electronic noise in the image chain (6). This improves the signal-to-noise ratio and may ultimately reduce the X-ray dose. It also improves the image quality to a certain extent, by compensating for a reduction in image quality due to the increased noise, which may reduce the radiation damage to patients. Consequently, 256-slice spiral CT may be used in the study of newborns with suspected HIE. The results of the current study demonstrated that there was no diagnostic difference in the image quality between low dose and conventional dose CT scanning (P>0.05).

The higher the applied X-ray dose used during a CT scan, the greater the likelihood of radiation damage. A previous study demonstrated that after receiving the same radiation dose, the risk of brain tumors and leukemia in children is much higher compared with that in adults, and younger children have a greater risk (7). Due to the specificity of neonatal physiology, children are likely to be exposed to larger doses and potentially develop more radiation damage than adults under the same scanning conditions. Therefore, the parameters of CT examination for children and newborns should be adjusted accordingly. Also, a smaller field of view and collimator should be used (8). Reducing the radiation dose in CT scanning is the main goal in protecting the neonatal brain. In the present study, the DLP in the lowest dose group was ~80% lower than that in the standard dose group (60.2 vs. 311.6 mGy). The CT images were all of diagnostic quality and the newborn brain was protected. In summary, a 256-slice CT scan using a lower radiation dose may be used to safely screen the neonatal brain without a reduction in the overall image quality.

## Figures and Tables

**Table I:** Parameters and results of computed tomography (CT) scanning.

Group	kV	mAsec	CTDI (mGy.cm)	DLP (mGy)	Signal noise (HU)	Image quality (mean ± SD)
Standard dose	120	250	30.4	311.6	1.78±0.42	2.55±0.29
Low dose group 1	120	150	24.8	109.7	1.95±0.35	2.25±0.41
Low dose group 2	120	50	6.2	60.2	2.36±0.49	2.05±0.74

CTDI, computed tomography dose-weighted index; DLP, dose-length product; SD, standard deviation.

## References

[1] McKinney AM, Teksam M, Felice R (2004). Diffusion-weighted imaging in the setting of diffuse cortical laminar necrosis and hypoxic-ischemic encephalopathy. AJNR Am J Neuroradiol.

[2] Fee SC, Malee K, Deddish R (1990). Severe acidosis and subsequent neurologic status. Am J Obstet Gynecol.

[3] Ferrari F, Todeschini A, Guidotti I (2011). General movements in full-term infants with perinatal asphyxia are related to Basal Ganglia and thalamic lesions. J Pediatr.

[4] Berrington de González A, Mahesh M, Kim KP (2009). Projected cancer risks from computed tomographic scans performed in the United States in 2007. Arch Int Med.

[5] Mori S, Endo M, Nishizawa K (2006). Comparison of patient doses in 256-slice CT and 16-slice CT scanners. Br J Radiol.

[6] Endo M, Mori S, Tsunoo T, Miyazaki H (2006). Magnitude and effects of x-ray scatter in a 256-slice CT scanner. Med Phys.

[7] Pearce MS, Salotti JA, Little MP (2012). Radiation exposure from CT scans in childhood and subsequent risk of leukaemia and brain tumours: a retrospective cohort study. Lancet.

[8] Strauss KJ, Goske MJ, Kaste SC (2010). Image gently: ten steps you can take to optimize image quality and lower CT dose for pediatric patients. AJR Am J Roentgenol.

